# Structural and immunoendocrine remodeling in gut, pancreas and thymus in weaning rats fed powdered milk diets rich in Maillard reactants

**DOI:** 10.1038/s41598-022-08001-w

**Published:** 2022-03-08

**Authors:** J. Dereke, E. Ekblad, B. Weström, C. Erlanson-Albertsson, M. Landin-Olsson, I. Sjöholm, M. Hillman

**Affiliations:** 1 Department of Clinical Sciences, Faculty of Medicine, Lund University, Lund, Sweden; 2 Neurogastroenterology Unit, Department of Experimental Medical Science, Faculty of Medicine, Lund University, BMC, B11, Sölvegatan 19, 22184 Lund, Sweden; 3 Department of Biology, Faculty of Science, Lund University, Lund, Sweden; 4 Department of Food Technology, Engineering and Nutrition, Faculty of Engineering, Lund University, Lund, Sweden

**Keywords:** Immunology, Physiology, Endocrinology, Gastroenterology

## Abstract

Western diet is extending worldwide and suspected to be associated with various metabolic diseases. Many food products have skim milk powder added to it and, during processing, lactose reacts with milk proteins and Maillard reaction products (MRPs) are formed. Dietary MRPs are suggested risk factors for metabolic dysregulation, but the mechanisms behind are still enigmatic. Here we describe that weaning rats fed diets rich in MRPs are affected in both their immune and endocrine systems. Marked structural changes in pancreas, intestine and thymus are noted already after 1 week of exposure. The pancreatic islets become sparser, the intestinal mucosa is thinner, and thymus displays increased apoptosis and atrophy. Glucagon- like peptide-1 (GLP-1) seems to play a key role in that the number of GLP-1 expressing cells is up-regulated in endocrine pancreas but down-regulated in the intestinal mucosa. Further, intestinal GLP-1-immunoreactive cells are juxta positioned not only to nerve fibres and tuft cells, as previously described, but also to intraepithelial CD3 positive T cells, rendering them a strategic location in metabolic regulation. Our results suggest dietary MRPs to cause metabolic disorders, dysregulation of intestinal GLP-1- immunoreactive cells, arrest in pancreas development and thymus atrophy.

## Introduction

Western diet is rich in advanced glycation end products (AGE) recognized to cause metabolic dysregulation^1,2^. Maillard reaction products (MRP) are AGEs present in high amounts in processed food. They are non-enzymatically formed by linkage of carbohydrates and peptides during heating and modified during storage. High dietary intake of MRPs has been linked to the development of several chronic pathologies like diabetes and its complications, and autoimmunity^3^. The pathophysiological mechanisms behind MRPs negative health effects are at present unclear. Two main paths are discussed^4^. Immune related effects including chronic intestinal inflammation, enhanced antigen presentation and increased oxidative stress is one. The other involves malnutrition and protein digestibility and, low bioavailability of lysine and minerals like iron and calcium.

Noteworthy is that infant formula milk is rich in MRPs, particularly carboxy-methyl lysine (CML), and is suspected to render the infant susceptible to inflammatory diseases and hypersensitivity^2^. Feeding weaning rats on a diet based on skim milk powder with a high content of MRP for a few weeks results in reduced body and thymus weights and increased inflammatory cytokines in the systemic circulation^5^. Since the intestinal barrier appeared intact and no faecal calprotectin increase was detected, intestinal inflammation as the primary pathophysiological mechanism behind is questioned. However, more detailed studies are needed for thorough knowledge on MRP- induced disease hazards. Postnatal thymus atrophy is a well-known effect of both malnutrition and inflammation^6,7^ and is attributed to lack of growth factors. Food intake stimulates the release of several intestinal hormones, like glucose-dependent insulinotropic polypeptide (GIP), glucagon-like peptide-1 (GLP-1), GLP-2 and peptide YY (PYY). Many which act as, or cause release of, growth factors^8,9^. Together these findings suggest a complex inter-play between the immune and the endocrine systems jointly shaping the internal metabolic milieu. Considering the health hazards connected with Western diet, especially in the young, the metabolic effects of dietary AGE/MRPs need to be further explored.

Aim of this study was to examine the consequences of dietary MRPs on digestive and immune organs by feeding weaning rats pelleted spray dried milk with three different levels of induced Maillard reactions; control skim milk powder (C) or skim milk powder stored at high temperature and humidity for 2 weeks, here termed medium Maillard (MM), or 4 weeks, here termed high Maillard (HM), skim milk powder. Possible changes in body weight gain, visceral organ weights and, circulating insulin, insulin growth factor-1 (IGF-1) and leptin levels were examined. Further, possible remodeling or histopathological changes e.g., inflammation in thymus, spleen, pancreas, and small and large intestine, or changes in the distribution and hormone expressions of pancreatic islets and enteroendocrine GLP-1 cells were performed using morphometry and immunocytochemistry.

## Results

### General observations, body and organ weights

**Figure 1:**
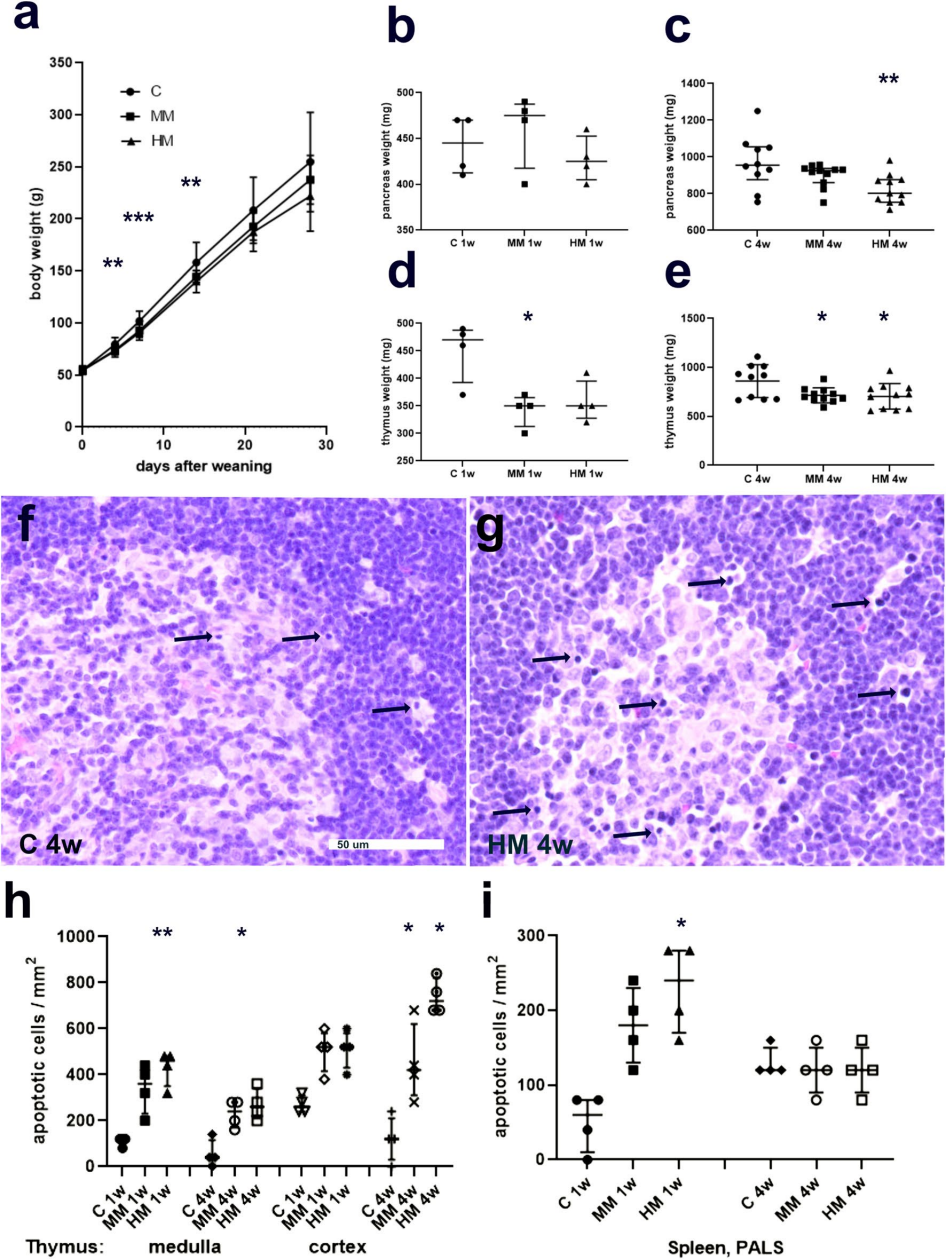
Body weights, thymus and pancreas weights and apoptosis in thymus and spleen in rats fed control (C), medium Maillard (MM)- and high Maillard (HM)-containing diets from weaning and start of experiment (day 0) to day 28. (**a**) Body weight (g) is presented as means ± SD, n = 14–14 (0–7 days) and 10–11 (14–28 days). (**b**,**c**) Pancreas and (**d**,**e**) thymus weights (mg) after 1 week (1w) and 4 weeks (4w). Data is in (**b**–**d**) presented as median (interquartile range) and in (**e**) as means ± SD; (b and d) n = 4, (**c**,**e**) n = 10–11. (**f**,**g**) Htx-E- stained sections of rat thymus after 4 weeks on (**f**) C diet (C 4w) or (**g**) HM diet (HM 4w). Increased number of pyknotic/apoptotic lymphocytes (exemplified with arrows) is noted after the HM diet. Scale bar is 50 μm and applies to both (**f**,**g**). (**h**,**i**) Cell counting revealed increased numbers of apoptotic lymphocytes in both thymic medulla and cortex (**h**), as well as in periarteriol lymphoid sheats (PALS) in the spleen (**i**). Data presented as median (interquartile range), n = 4 for rats on diet for 1w and n = 10–11 for 4w. Comparisons between groups were done with Kruskal–Wallis followed by Dunn’s multiple comparison test or one- way ANOVA followed by Dunnett’s multiple comparison test; *p < 0.05, **p < 0.01, ***p < 0.001.

All rats appeared healthy and exhibited normal activity throughout the study period, and so all animals were included in the study. Rats fed the MM or HM diets for 2 weeks gained less weight than rats on the C diet despite equal feed intake. After 4 weeks no differences in body weight or feed intake were found (Fig. 1a and supplementary Tables 1S and 2S).

At autopsy, the visceral organs were inspected, and no lesions or abnormalities were identified. Rats fed the MM diet for 1 and 4 weeks displayed lower thymus weight while no weight differences were noted in spleen, pancreas, or small and large intestine, compared to rats fed the C diet. Rats fed the HM diet for 1 week transiently showed a lower large intestinal weight; after 4 weeks the organ weights of pancreas and thymus were reduced while no weight differences were noted in spleen or intestine compared to rats fed the C diet. Results are summarised in Fig. 1b–e and Table 1.

**Table 1:** Weights (mg) of organs from rats weaned at 3 weeks of age and fed control (C), medium Maillard (MM) or high Maillard (HM) containing diets for 1 or 4 weeks.

	C	MM	p	HM	p
**1 week**					
Thymus	470.0 (392.5–487.5)	350.0 (321.5–365.0)	0.0429	350.0 (327.5–395.0)	ns
Spleen	597.5 (533.8–650.0)	450.0 (415.0–560.0)	ns	440.0 (387.5–515.0)	ns
Pancreas	445.0 (412.5–470.0)	475.0 (417.5–490.0)	ns	425.0 (425.0–452.5)	ns
Small intestine	6,180.0 (5,288.0–6,788.0)	5,935.0 (5,793.0–6,460.0)	ns	6,440.0 (5,815.0–6,878.0)	ns
Large intestine	1,010.0 (897.5–1,078.0)	840.0 (690.0–907.5)	ns	805.0 (755.0–840.0)	0.0278
**4 weeks**					
Thymus	860.6 ± 168.1	715.0 ± 76.5	0.0278	704.9 ± 130.1	0.0183
Spleen	873.5 (699.3–1054.0)	835.0 (771.0–884.0)	ns	817.0 (612.0–950.0)	ns
Pancreas	955.0 (876.0–1056.0)	925.0 (860.0–937.0)	ns	802.0 (753.0–876.0)	0.0067
Small intestine	9,164.0 ± 1,653.0	8,524.0 ± 1,251.0	ns	8,040.0 ± 1,294.0	ns
Large intestine	1,521.0 ± 243.6	1,488.0 ± 195.6	ns	1,323.0 ± 180.4	ns

Values from rats fed 1 week are expressed as median (interquartile range), n = 4. Kruskal–Wallis followed by Dunn´s multiple comparison test. Values from rats fed 4 weeks are expressed as mean ± SD or median (interquartile range), n = 10–11. One-way ANOVA followed by Dunnett´s multiple comparison test or Kruskal–Wallis followed by Dunn´s multiple comparison test), p = p value as compared to C, ns = no significant differences compared to C.

### Morphology, morphometry, and immunocytochemistry

#### Thymus and spleen

Htx-E stained sections revealed a normal general histology in all rats, irrespective of diet. The thymic medulla-cortex delineation was well defined, and no bleedings, fat cell infiltration or other anomalies could be identified. However, a marked increase in the number of apoptotic cells, identified as pyknotic cells, were noted in both the medullary and cortical regions in animals fed MM or HM, compared to C diet (Fig. 1f–h). In spleen the red pulp contained splenic cords and in between erythrocytes, immune and hematopoietic cells. The white pulp was well defined with periarteriol lymphoid sheats (PALS), follicles and marginal zones. Apoptotic cells were counted in PALS and found to be increased in number in rats subjected to HM diet for 1 week. After 4 weeks no differences in number of apoptotic cells were found in the different groups (Fig. 1i).

#### Pancreas

**Figure 2:**
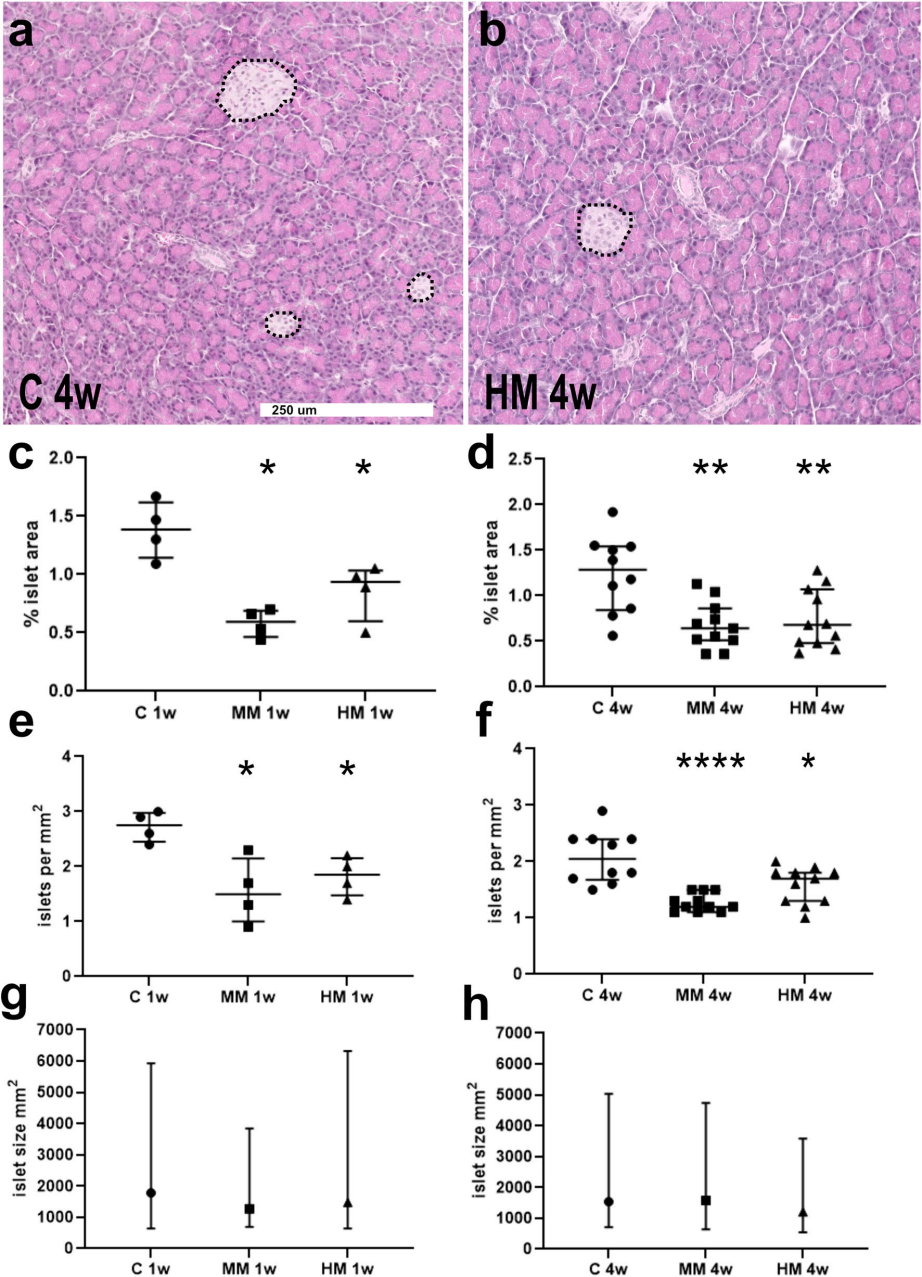
Morphometric measurements of endocrine tissue on Htx-E-stained sections of pancreas from rats fed control (C), medium Maillard (MM)- and high Maillard (HM)-containing diets for 1 (1w) and 4 weeks (4w). Pancreatic sections after 4 weeks fed (**a**) control (C 4w) or (**b**) high Maillard-containing (HM 4w) diets. Islets of endocrine cells (marked with dotted lines) are interspersed within the exocrine pancreatic tissue. Scale bar is 250 μm and applies to both a and b. (**c**,**d**) The area of islets, expressed as % of total area of pancreatic tissue, was measured on Htx-E-stained sections. This revealed a lower proportion of the endocrine area in rats fed MM and HM diets, compared to the C diet. This was mainly due to a lower number of islets per mm^2^ pancreatic tissue as shown in (**e**,**f**) and not reduced islet size (**g**,**h**). Rats on diets for 1w: n = 4, for 4w: n = 10–11. Data presented as medians (interquartile range). Comparison between groups were done with Kruskal–Wallis followed by Dunn’s multiple comparison test; *p < 0.05, **p < 0.01, ***p < 0.001. (**g**,**h**) The islet sizes presented as medians (interquartile range), were found to vary greatly, but median size did not differ between the different diet groups (Kruskal–Wallis test); C 1w, n = 175; MM 1w, n = 108; HM 1w, n = 144; C 4w, n = 386; MM 4w, n = 263 and HM 4w, n = 352.

Htx-E stained sections from pancreas revealed a normal general histology of both the exo- and endocrine parts in all rats irrespective of diet. No inflammation, fibrosis, or bleedings were found. Morphometric measurements revealed a marked decrease, about halved, of the proportion of the endocrine component in relation to the entire pancreatic tissue area in rats fed MM and HM diets, for 1 and 4 weeks, compared to rats fed C diet (Fig. 2a–d). To examine if this difference was the result of fewer and/or smaller islets, compared to C rats, the numbers and sizes of islets were estimated. This revealed that the total numbers of islets per mm^2^ pancreatic tissue were lower in MM and HM rats (Fig. 2e,f) while the median islet size did not differ in MM and HM rats, compared to C rats (Fig. 2g,h). Immunocytochemistry visualizing insulin-, glucagon-, GLP-1-, and somatostatin-immunoreactive (IR) cells showed small-as well as large-sized endocrine pancreatic islets uniformly distributed throughout the pancreas. Insulin-IR cells occupied the central parts of the islets while glucagon-, GLP-1-, and somatostatin-IR cells were located peripherally. Single or small clusters of extra-insular endocrine cells of all classes were commonly noted.

**Figure 3:**
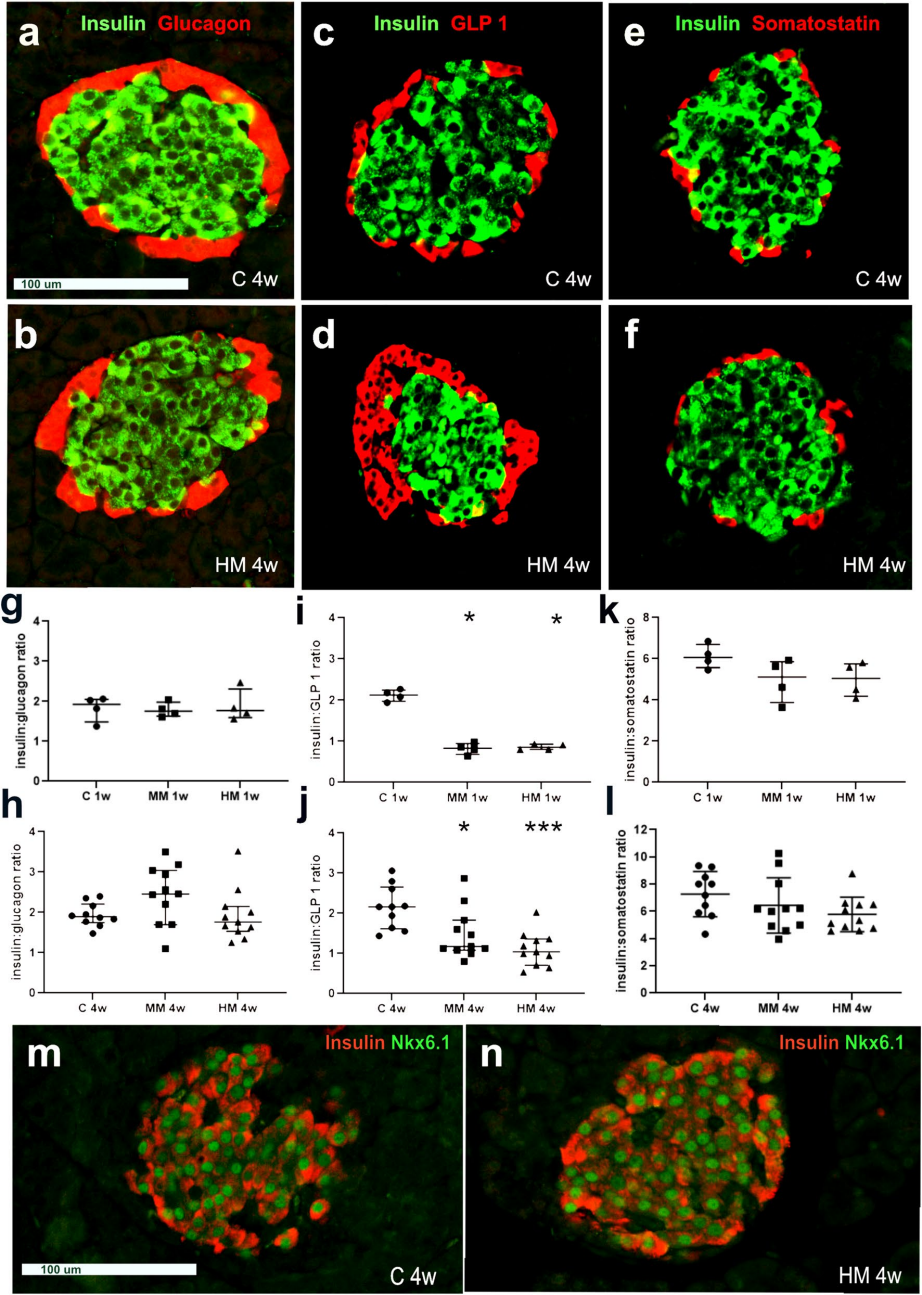
Insulin-, glucagon-, glucagon-like peptide-1 (GLP-1)-, and somatostatin- immunoreactive (IR) endocrine cells in pancreas from rats fed control (C), medium Maillard (MM)- and high Maillard (HM)-containing diets for 1 (1w) and 4 weeks (4w). (**a**–**f**) Pancreatic sections from rats after 4 weeks fed C (C 4w) or HM (HM 4w) diets were double immunostained for (**a**,**b**), insulin (green) and glucagon (red), (**c**,**d**) insulin (green) and GLP-1 (red), and (**e**,**f**) insulin (green) and somatostatin (red). The topographic distribution of endocrine cells, with central insulin-IR cells and peripherally located glucagon-, GLP-1- and somatostatin-IR cells is similar in all rats irrespective of diet. Notable is, however, that the number of GLP-1-IR cells is higher in the rat fed HM 4w (**d**), as compared to (**c**)**,** the C 4w rat. Scale bar is 100 μm and applies to (**a**–**f**). (**g**,**h**) Cell counting revealed that the insulin:glucagon cell ratios were approximately 2 in all rats irrespective of if they were fed C, MM- or HM-containing diets for 1 week (**g**) or 4 weeks (**h**). (**i**,**j**) The insulin:GLP-1 cell ratios were approximately 1 in rats fed MM or HM diets at both 1 week (1w) and 4 weeks (4w), which was markedly lower than, approximately 2, in pancreas from C rats. (**k**,**l**) The insulin:somatostatin cell ratios were similar in all rats irrespective of diet. (**m**,**n**) Pancreatic sections double immunostained for insulin (red) and the homeobox protein Nkx6.1 (green) from rats fed C or HM diets for 4 weeks (4w). Note the selective nuclear expression of Nkx6.1 protein in all insulin-IR cells. Scale bar is 100 μm and applies to both m and n.

The general topographic distribution of pancreatic endocrine cells appeared similar in all groups of rats (Fig. 3a–f). Immunocytochemical staining against the pan T-cell marker revealed scattered CD3-IR cells in the pancreatic stroma but none in pancreatic islets in all rats, irrespective of diets.

Double immunolabelling and cell counting were used to reveal the ratio of insulin:glucagon-, insulin:GLP-1-, and insulin:somatostatin-IR cells in the different diet groups of rats. These examinations revealed that the insulin:glucagon cell ratios were approx. 2:1 in C as well as in MM and HM exposed rats (Fig. 3g,h). Insulin:GLP-1 cell ratios were approx. 2:1 in C rats, while rats fed MM and HM diets all showed insulin:GLP-1 cell ratios of approx. 1:1 at both 1 and 4 weeks (Fig. 3i,j). Insulin:somatostatin-IR cell ratios were approx. 6:1 in rats treated for 1 week, irrespective of diet, and in the range of 7:1 to 9:1 in rats treated for 4 weeks (Fig. 3k,l). No significant differences between the MM or HM rats, compared to C rats, could be determined.

Islet cells containing both insulin and glucagon were extremely few but could occasionally be found in all groups of rats irrespective of diet. Insulin-IR cells were never found to be GLP-1- or somatostatin-IR. Double immunolabelling of insulin and Nkx6.1 or PDX-1 revealed all insulin-IR cells also to be Nkx6.1- and PDX-1-IR; no Nkx6.1- or PDX-1-IR cells were found to lack insulin (Fig. 3m,n).

#### Proximal and distal small intestine and proximal large intestine

The general histology of the intestines appeared normal in all rats irrespective of dietary group and no inflammatory reactions, bleedings or mucosal shedding were noted. Morphometry on Htx-E stained sections from proximal and distal small intestine and proximal large intestine from rats fed the C, MM, or HM diets for 1 week revealed no remodelling in terms of intestinal wall thickness or of the various intestinal layers (mucosa, submucosa and muscularis propria). Rats fed MM and HM diets for 4 weeks displayed markedly reduced mucosa thicknesses in proximal and distal small intestine, but not in proximal large intestine, compared to rats on C diet. The intestinal submucosa and muscularis propria layers were equally thick in all rats in the 4 weeks ´ groups, irrespective of diet. The results are summarized in Table 2.

**Table 2:** Morphometric analysis of whole wall and layer thickness (µm) in proximal and distal small (SI) and proximal large intestine (LI) as estimated on Htx-E stained sections from rats fed control (C), medium Maillard (MM) or high Maillard (HM) containing diets for 1 or 4 weeks.

		Whole wall	p	Mucosa	p	Submucosa	p	Muscularis propria	p
**1 week**									
Proximal SI	C	693.0 (658.0–731.8)		609.0 (570.0–643.0)		17.0 (14.5–18.8)		71.0 (61.5–76.0)	
	MM	661.5 (648.5–687.3)	ns	591.5 (566.0–602.0)	ns	15.5 (13.5–16.75)	ns	69.0 (59.3–81.0)	ns
	HM	670.0 (635.5–739.8)	ns	588.0 (565.0–637.3)	ns	17.5 (16.3–18.8)	ns	70.0 (55.5–81.0)	ns
Distal SI	C	486.5 (484,0–516.8)		429.0 (413.0–448.8)		12.0 (11.3–13.5)		54.0 (48.8–61.5)	
	MM	492.5 (444.5–572.0)	ns	428.5 (382.8–486.3)	ns	13.0 (11.3–16.8)	ns	48.0 (43.5–65.3)	ns
	HM	519.5 (460.3–571.3)	ns	439.5 (395.5–498.5)	ns	14.0 (12.0–16.8)	ns	56.0 (50.0–73.3)	ns
Proximal LI	C	469.5 (396.8–589.5)		246.5 (234.3–305.3)		45.5 (35.0–71.7)		184.0 (134.8–222.8)	
	MM	425.0 (391.8–427.5)	ns	229.0 (219.3–254.5)	ns	30.0 (22.3–37.0)	ns	150.5 (136.0–165.8)	ns
	HM	433.0 (403.5–443.0)	ns	242.0 (230.0–248.0)	ns	29.5 (26.8–31.5)	ns	162.0 (144.0–165.0)	ns
**4 weeks**									
Proximal SI	C	818.4 ± 51.7		715.4 ± 55.4		20.5 ± 4.1		77.1 ± 10.2	
	MM	736.6 ± 71.8)	0.0172	634.9 ± 59.8	0.0097	19.3 ± 3.0	ns	77.7 ± 18.6	ns
	HM	686.9 ± 74	0.0002	601.8 ± 66.5	0.0004	17.9 ± 2.7	ns	68.3 ± 10.1	ns
Distal SI	C	592.0 ± 69.9		507.9 ± 63.4		19.4 ± 2.5		59 ± 6.8	
	MM	500.0 ± 06.5	0.0266	428.7 ± 84.7	0.0249	17.6 ± 3.1	ns	53.1 ± 13.6	ns
	HM	485.6 ± 57.3	0.0102	410.3 ± 54.3	0.0056	16.9 ± 1.7	ns	52.7 ± 9.6	ns
Proximal LI	C	414.9 ± 49.3		215.5 ± 24.0		29.1 ± 2.1		170.7 ± 38.7	
	MM	420.5 ± 35.0	ns	213.6 ± 13.3	ns	26.6 ± 6.7	ns	179.8 ± 25.7	ns
	HM	411.2 ± 35.8	ns	210.9 ± 18.5	ns	26.4 ± 3.9	ns	174.5 ± 19.4	ns

Values from rats fed 1 week are expressed as median (interquartile range), n = 4, Kruskal–Wallis followed by Dunn´s multiple comparison test), Values from rats fed 4 weeks are expressed as mean ± SD, n = 10–11, one-way ANOVA followed by Dunnett´s multiple comparison test. *P* p value as compared to C, *ns* no significant differences compared to C.

**Figure 4:**
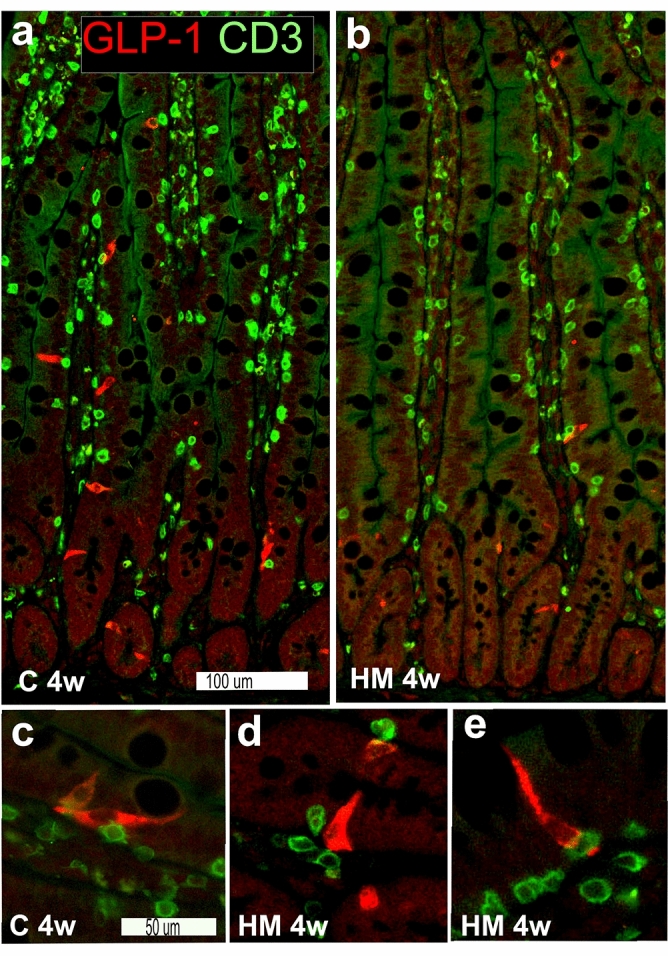
Topographic distribution and proximity of glucagon-like peptide-1 (GLP-1)- (red) and CD3-IR (green) intestinal cells in sections from distal small intestine from rats fed control (C) or high Maillard (HM) diets for 4 weeks (4w) (**a**,**b**). GLP-1-IR cells are regularly found in both villi and crypt epithelium, particular in C diet rats. CD3-IR cells are numerous throughout the mucosa, in lamina propria as well as intraepithelial, in both C (**a**) and HM (**b**) diet rats. Scale bar is 100 μm and applies to a and b. Note frequent close contacts between GLP-1- and CD3-IR cells as shown in higher magnification in both C (**c**) and HM (**d**,**e**) diet rats. Scale bar in (**c**) is 50 μm and applies to (**c**–**e**).

Immunocytochemistry against the pan T-cell marker CD3 showed numerous CD3-IR cells in the intestinal mucosa, particularly in lamina propria but also intraepithelial (Fig. 4a,b). CD3-IR cells were occasionally found also in the submucosa but they were extremely few in muscularis propria. The distribution and frequency of CD3-IR cells in distal small or proximal large intestine did not differ between C, MM and HM rats. Immunocytochemistry against GLP-1 revealed GLP-1-IR endocrine cells scattered in the epithelium of both distal small (Fig. 4a,b) and proximal large intestine in all rats, irrespective of dietary group.

Double immunostaining and cell counting of CD3- and GLP-1-IR cells were used to estimate the numbers of intraepithelial CD3-IR T cells and GLP-1-IR endocrine cells and their possible contacts, in terms of being in proximity, within the intestinal epithelia (Fig. 4, Table 3). In villi of the distal small intestine 25–30 CD3-IR cells/mm epithelium were found in all rats irrespective of experimental group. The numbers of GLP-1-IR cells were 2–3/mm epithelium in rats fed 1 week on C, MM or HM diets and in rats fed 4 weeks on C diet, while in rats fed 4 weeks on MM or HM diets the numbers of GLP-1-IR cells were significantly reduced. Approximately 40% of all GLP-1-IR cells were in close contact to CD3-IR cells. In distal small intestinal crypts, the numbers of CD3-IR cells/mm epithelium were approximately 5 in all rats irrespective of experimental group. The numbers of GLP-1-IR cells were 3–5/mm epithelium in rats subjected to 1 week of C, MM or HM diets and in rats subjected to 4 weeks of C or MM diets, while in rats subjected to 4 weeks of HM diet the number of GLP-1-IR cells was significantly reduced. Approximately 5% of all GLP-1-IR cells were in close contact to CD3-IR cells. In proximal large intestinal surface epithelia, the numbers of CD3-IR cells/mm epithelium were approximately 15 in all rats subjected to 1 week of C, MM and HM diets and 6–7 in all rats subjected to 4 weeks of C, MM and HM diets. The numbers of GLP-1- IR cells were 5–7/mm epithelium in rats subjected to 1 week of C, MM and HM diets and in rats subjected to 4 weeks of C diet, while in rats subjected to 4 weeks of MM or HM diets the numbers of GLP-1-IR cells were significantly reduced. Approximately 10% of all GLP-1-IR cells were in close contact to CD3-IR cells. In proximal large intestinal crypts, the numbers of CD3-IR cells/mm epithelium were 2–4 in all rats irrespective of dietary group. The numbers of GLP-1-IR cells were 5–7/mm epithelium in rats fed 1 week of C, MM and HM diets and in rats fed 4 weeks of C diet, while in rats fed 4 weeks on MM or HM diets the numbers of GLP- 1-IR cells were significantly reduced. Only few GLP-1-IR cells were in close contact to CD3- IR cells.

**Table 3:** Numbers of glucagon-like peptide-1 (GLP-1)- and CD3-immunoreactive (IR) cells per mm intestinal epithelium and relative frequency of GLP-1-IR cells in close contact with CD3-IR cells in villi and crypts from distal small intestine (dSI) and surface epithelium (epi) and crypts from proximal large intestine (pLI) as estimated on immunostained sections from rats fed control (C), medium Maillard (MM) or high Maillard (HM) containing diets for 1 or 4 weeks.Values from rats fed 1 week (n = 4) or 4 weeks (n = 10–11) expressed as median (interquartile range).

	GLP-1-IR cells/mm	p	CD3-IR cells/mm	p	% GLP-1-IR cells in contact with CD3 -IR cells	p
**1 week**						
**dSI, villi**						
C	2.1 (1.6–2.7)		26.3 (22.9–37.0)		37.9 (17.3–47.5)	
MM	2.3 (2.0–2.7)	ns	29.3 (25.6–30.9)	ns	32.6 (25.3–49.2)	ns
HM	3.9 (2.1–5.8)	ns	27.6 (24.7–31.6)	ns	39.6 (31.9–46.5)	ns
**dSI, crypts**						
C	4.5 (3.3–5.4)		3.8 (3.7–4.1)		7.5 (5.4–12.1)	
MM	4.7 (4.3–5.6)	ns	5.0 (4.4–5.5)	ns	4.4 (3.7–11.3)	ns
HM	4.6 (4.4–5.6)	ns	6.2 (3.9–6.7)	ns	1.9 (0–13.9)	ns
**pLI, epi**						
C	4.6 (3.9–6.0)		12.6 (9.8–23.3)		16.1 (13.3–24.2)	
MM	7.1 (4.9–9.2)	ns	13.5 (11.1–18.3)	ns	8.0 (4.2–18.7)	ns
HM	5.2 (4.5–7.8)	ns	17.8 (16.4–19.0)	ns	12.6 (5.6–13.9)	ns
**pLI, crypts**						
C	5.1 (3.3–6.2)		4.2 (2.4–6.6)		7.4 (4.1–14.8)	
MM	5.2 (4.9–5.8)	ns	2.5 (1.0–2.9)	ns	11.0 (1.2–19.0)	ns
HM	4.7 (3.6–5.5)	ns	2.8 (2.0–4.1)	ns	0 (0–4.9)	ns
**4 weeks**						
**dSI, villi**						
C	2.3 (2.0–2.6)		26.5 (21.4–29.6)		43.2 (37.5–47.0)	
MM	1.3 (1.1–1.5)	0.0068	22.9 (20.8–36.7)	ns	44.0 (36.8–47.9)	ns
HM	1.5 (1.1–2.0)	0.0453	27.2 (22.5–31.8)	ns	32.2 (15.3–39)	ns
**dSI, crypts**						
C	3.1 (2.4–3.8)		5.1 (4.1–6.8)		7.5 (5.7–12.6)	
MM	1.8 (1.0–2.7)	ns	4.2 (3.7–5.0)	ns	0 (0–15.5)	ns
HM	1:4 (0.8–1.9)	0.0068	3.3 (2.5–5.0)	ns	5.0 (0–14.4)	ns
**pLI, epi**						
C	7.0 (5.6–9.3)		7.5 (6.3–8.6)		6.4 (3.3–9.9)	
MM	3.3 (1.9–4.1)	0.0116	6.9 (5.6–11.3)	ns	2.4 (0–8.7)	ns
HM	2.8 (2.3–3.6)	0.0070	6.0 (5.3–7.2)	ns	8.0 (7.0–19.9)	ns
**pLI, crypts**						
C	4.9 (4.5–5.2)		1.9 (0.9–2.6)		0 (0–4.4)	
MM	2.5 (1.3–2.9)	0.0053	1.8 (1.3–2.8)	ns	0 (0–2.1)	ns
HM	2.5 (2.1–2.8)	0.0089	2.7 (1.9–3.2)	ns	0 (0–4.3)	ns

Kruskal–Wallis followed by Dunn´s multiple comparison test), *p* p-value as compared to C, *ns* no significant differences compared to C.

### Blood and faecal analysis

Plasma levels of insulin, C-peptide, leptin and IGF-1 levels were investigated using ELISA. No statistically significant differences in insulin, C-peptide or leptin levels could be observed between the different dietary groups of rats after either 1- or 4-weeks. IGF-1 levels were significantly decreased in the MM group, compared to the C group after 1 week of experimentation. After 4 weeks no differences in IGF-1 levels were noted between the three groups. Results are summarised in supplementary Tables 3S and 4S.

Faecal calprotectin levels were investigated using ELISA and found to be extremely low or below detection in all rats irrespective of diet (supplementary Table 4S).

### Intestinal permeability

After 1 week of dietary exposure intestinal permeability was tested by gavage of the different- sized markers, FITC-dextan 4000 and albumin. No differences in the serum levels were found, 4 h after administration of the marker gavage, between the dietary groups (supplementary Table 4S and 5S).

## Discussion

Feeding weaning rats on a milk powder-based diet with high MRP content for 1 and 4 weeks resulted in marked histopathological changes in thymus, pancreas, and intestines. The body weight gain initially slowed down, but after 4 weeks no differences in body weights were noted.

The thymus weight was substantially reduced in both MM and HM rats, and thymus contained increased numbers of apoptotic cells in both cortex and medulla. Postnatal thymus atrophy is a well-known effect of malnutrition in both man^6,7^ and rat^10^, and low IGF-1 level is considered a reliable marker in malnutrition^11^. Diets containing high levels of MRP are reported to have reduced digestibility and nutrient availability^3,12^ which may provide a basis for malnutrition in the present study. The reduction of body weight gain, thymus and pancreas weights, small intestinal mucosal thickness and IGF-1 plasma levels in rats exposed to MRP-rich diets, as found in the present study, speak in favor of malnutrition and immune dysfunction.

Dietary AGEs formed by the Maillard reaction during food processing have been associated with changed glucose homeostasis, inflammation, and oxidative stress as well as endothelial and renal functions^1,3^. Infant formula based on milk powder is a rich source of MRPs, particularly CML, rendering the infant prone to inflammation and hypersensitivity^2^. However, health effects of AGEs in man are difficult to study due to several methodological limitations^1^. Animal studies on pets and livestock consuming processed feed put forward MRPs to increase diet-related chronic gut inflammation^4,13^. The present study on rats fed diets high in MRPs failed to identify any signs of gut inflammation, as based on histopathological analysis and absence of calprotectin in faeces. Still, immune related effects leading to changes in e.g., antigen presentation or increased oxidative stress cannot be excluded.

The pancreas weight was markedly reduced in the 4 weeks HM fed rats. The proportions of pancreatic endocrine tissue (islets) were reduced by approximately 50% in rats fed MM and HM diets after 1 and 4 weeks. The numbers of insulin-, glucagon- and somatostatin-containing islet cells were all affected, as shown by the similar insulin:glucagon and insulin:somatostatin cell ratios in C- versus MM- and HM-fed rats. However, the insulin:GLP-1 cell ratios markedly decreased in rats on MM and HM diets already after 1 week and remained after 4 weeks, suggesting a relative increase in the number of GLP-1-expressing islet cells due to the MRP-rich diets. No concentration-dependent responses between the MM and HM diets were noted which indicate that CML/MRP-induced effects are reached already at low-grade exposure. The main mechanism behind loss of pancreatic endocrine tissue and increased numbers of GLP-1-IR islet cells probably reflect alterations in the interplay between metabolic, endocrine, and immunologic signaling.

The most intriguing findings in the present study are the early loss of pancreatic islets and the striking increase of GLP-1-IR cells in the peripheral parts of the islets already after 1 week of exposure to MRP-rich diets. The rat endocrine pancreas undergoes prominent changes after birth during the weeks up to weaning^14^. A wave of beta-cell apoptosis is met by a wave of neogenesis by the time of weaning, while replication, which is high perinatally, declines gradually and is low at weaning. The use of weaning rats, as done in the present study, suggest that exposure to dietary MRP may interfere with the ongoing development of endocrine pancreas. Transcription factors and active gene regulation are the basic determinants of pancreas development and maintenance^15^, while vast data indicate malnutrition, metabolic dysregulation, immune reactivity, or toxic substances as important determinants of endocrine pancreas plasticity. The exact role of each of these aspects and how they are affected by dietary MRPs are unknown. The apoptotic wave occurring around weaning is suggested an important trigger in causing autoimmunity and islet beta cell destruction in that it releases more antigen^16^. CML exposure is reported to cause beta cell damage and reduced insulin secretion due to RAGE upregulation, mitochondrial dysfunction and mitophagy of pancreatic beta cells^17^. Our results indicate that dietary exposure of MRPs during weaning further increases the apoptotic wave causing imbalance between apoptosis and neogenesis and the net result would be loss of endocrine cell mass. If this in turn leads to autoimmunity and beta, and possibly also alpha and delta, cell destruction can only be speculated on. Thymic dysfunction may further aggravate islet autoimmunity^16^.

The here reported loss of pancreatic endocrine cells, including beta cells, may trigger regeneration including trans-differentiation of other mature pancreatic cells to beta cells by genetic reprogramming^18^. If conversion of alpha cells to beta cells, or vice versa, took place within the islets after exposure to MRP-rich diets were studied by double immunostaining. The results showed all insulin-IR cells to exclusively contain, the beta cell transcription factors, Nkx6.1 and PDX-1. Occasional islets cells contained both insulin and glucagon, in all groups of rats. Together this suggests that beta cell regeneration rates were low in all rats irrespective of diet at this age.

An interesting, but unexpected finding was that the relative numbers of GLP-1-IR islet cells markedly increased in rats on MRP-rich diets. These cells outnumbered the glucagon-IR cells, were non-immunoreactive to insulin and located in the periphery of the endocrine islets. Thus, our results suggest that pancreatic alpha cells constitute at least two subpopulations: one expressing both glucagon and GLP-1 and another GLP-1 only. The exact overlap of the glucagon- and the GLP-1-IR cells could not be determined since both our selective antisera were generated in rabbits, rendering double-immunostaining impossible to implement.

The present findings of a lower number of pancreatic islets in MRP-exposed rats strikingly resembles recent findings in patients with type 1 diabetes (T1D)^19^. Pancreatic biopsies from patients with recent onset or longstanding T1D showed a marked reduction in islet density but preserved islet size. In patients with T1D the islets displayed loss of insulin-IR cells but increased numbers of glucagon-IR cells^19^. The possible presence of pancreatic GLP-1-IR cells was not examined in the T1D patients.

The loss of beta cells and the marked up-regulation of GLP-1 containing islet cells in rats on MRP rich diets indicate that these rats are at risk to develop failing insulin secretion. Their circulating levels of insulin and C-peptide did, however, not deviate from C rats suggesting that the increased number of pancreatic GLP-1 expressing cells are able to act as an intra-islet incretin and compensate for the beta cell loss by increased stimulation of insulin release. For how long this will sustain is, however, an open question. The rats were followed for 4 weeks and long-term studies will be needed to determine future insulin synthesis and release, glucose homeostasis and possible regeneration of beta cells.

Coinciding with the increase in GLP-1-IR cells in the pancreas in rats fed diets with high MRP content we noted decreased numbers of intestinal GLP-1-IR (L) cells. Intestinal L cells are open endocrine cells stimulated by luminal nutrients to release the incretin GLP-1. One explanation for a lower number of L cells in rats on MRP-rich diets may be that the lower protein digestibility^3,12^ results in an attenuated stimulation of L cells and a low GLP-1 secretion. Thus, attenuated intestinal incretin stimulation may have caused the beta cell loss and the GLP-1-IR cells increase noted after MRP-rich diets. Intestinal GLP-1 secretion is, however, not only activated by luminal nutrients, also vagal and enteric neurons are suggested to contribute due to the close association of L cells and nerve terminals^8^. In the present study we found T cells (CD3-IR) and GLP-1-IR enteroendocrine cells preferentially juxta positioned. Along the distal small and proximal large intestines, the numbers of both GLP-1-IR cells and intraepithelial CD3-IR cells were low. However, it is notable that approximately 40% of all GLP-1-IR endocrine cells in small intestinal villi and up to 15% of the GLP-1-IR cells in large intestinal surface epithelia were in proximity with CD3-IR cells. No change in the numbers of GLP-1- and CD3-IR cells, or in the frequency of GLP-1 to CD3 cell contacts were noted between the different dietary groups of rats, probably indicating that anatomic disconnection between T and L cells is not the root cause of the lower numbers of L cells in rats on MRP-high diets. The substantial proportion of GLP-1- IR cells in strategic proximity with intraepithelial T cells provides, however, a morphological basis to suggest immune mechanisms to play a role in post-prandial signaling. In fact, intraepithelial T cells are shown to express GLP-1 receptors and to regulate systemic metabolism by controlling the bioavailability of GLP-1^20^. The functional significance of such immunoendocrine co-play needs further examination, also taking into consideration the close association of GLP-1-IR cells with nerve endings^8^ and tuft cells^21^.

Strengths of the study include the control of available lysine and CML levels induced in the milk-powders as well as the chow fed to the rats. This is not frequently reported in studies with dietary AGEs but is important when it comes to data interpretation and conclusions. The possibility to reliably distinguish between glucagon and GLP-1 in immunocytochemical staining is also considered a strength of the study.

Limitations of the study include that the 1-week feeding experiment only involved one litter with 4 rats (siblings) in each group which is underpowered, compared to the 4-week experiment. The inclusion of pups of both sexes with different growth rates might be considered a limitation. Not adding measurements of AEG in the various diets, using mass spectrometry, in addition to measuring CML levels by ELISA, may also be considered a limitation. Future studies will comprise detailed long-term studies to identify if rats on MRP rich diets will develop hypersensitivity or metabolic disorders as well as short-term studies to reveal the dynamics of the early MRP-induced changes in thymus and pancreas, and measurements of circulating GLP-1. In addition, it needs to be considered that degradation by the intestinal microbiota of dietary MRPs generates a lower amount of short chain fatty acids, as compared to control diets low in MRP^22^. This might cause dysbiosis and defect signaling between intestinal endocrine cells, immune cells, and gut microbiota. Thus, future studies will also focus on microbial flora diversity and possible cellular and topographic changes in enteroendocrine cells known to sense gut microbiota and regulate gut homeostasis^23^.

In conclusion: Western diet is extending worldwide and suspected to be associated with various metabolic diseases. Our results suggest dietary MRPs to cause nutritional disorder, dysregulation of intestinal GLP-1 cell contacts, arrest in pancreas development and thymus atrophy in a model with weanling rats.

## Methods

### Preparation of milk powder and chow

Pasteurized (+ 73 °C) and low heat spray dried cow skim milk powder (SM) was purchased from Falköpings Mejerier, Falköping, Sweden. Three SMs with different levels of MRPs were used for manufacturing rat feed pellets, the original purchased SM (control; C) and two after storage in a climate chamber at 45 °C and 52% relative humidity for 14 (medium Maillard skim milk powder; MM) and 28 days (high Maillard skim milk powder; HM)^24^.

The pelleted experimental diets were based on a laboratory rodent chow (Labfor R36, Lantmännen, Kimstad, Sweden) with C, MM and HM incorporated as the main protein source as previously described^5^.

Available lysine contents were determined using a dye-binding method^8,24^ and found to be 2.8 (C), 1.4 (MM) and 1.0 (HM) g per 100 g SM powder.

Measurements of N-epsilon-(carboxymethyl) lysine (CML) contents in the C, MM and HM powders and corresponding rodent pellets were performed using a commercial ELISA assay kit (ab238541, Abcam, Cambridge, UK) according to the manufacturer’s instructions.

The samples were diluted 1:2 (pellets) or 1:10 (SM) in assay diluent, absorbance measurement, in duplicates, at 450 nm using a FLOUstar Optima plate reader (BMG Labtech, Gmbh, Ortenburg, Germany). Unknown concentrations were estimated using a 10-point standard 4-parameter fit curve. The CML level was below detection level (< 0.05 μg/g) in the C powder, while elevated in the MM (30.5 μg/g) and the HM powders (79.7 μg/g). CML levels in the fed pellets were 3.4 μg/g in the C fed, 67.6 μg/g in the MM and 144.0 μg/g in the HM feds.

### Animals and study design

Pregnant Sprague–Dawley rats (n = 4, gestation day 14) were purchased (Janvier Labs, Saint Berthevin, France) and housed under specific pathogen free conditions in a climate- and light-controlled animal facility and a rat chow (Labfor R36, Lantmännen, Kinstad, Sweden) and water were supplied continuously. At 2–3 days after birth rat pups were culled to max 12 per litter and kept with their dam in separate cages equipped with a 7 cm wall extender to hinder access of solid chow by the pups, on aspen wood bedding (Beekay B&K Universal AB, Sweden) enriched with nesting material (Sizzle-nest, Scanbur, Denmark). All animal experiments were approved by the Lund/Malmö animal ethics committee, Lund, Sweden (diary no M169-14) and performed in accordance with the European Communities Council Directive (86/609/EEC and 2010/63/EU) and the Swedish Animal Welfare Act (SFS 1988:534). The study is planned, conducted and reported in accordance with ARRIVE guidelines.

At the age of 21 days (experimental day 0) the rat pups (n = 44) were separated from their dams and designated into three groups and fed one of two different experimental diets based on high Maillard reaction product content MM (n = 15), HM (n = 15) or the control diet C (n = 14). The rats were weighed in the morning using an electronic scale on days 0, 4, 7, 14, 21 and 28 while the feed consumption was measured weekly. After 1 week one litter with 4 rats from each experimental group was deprived of food and subjected to studies on intestinal permeability by oro- gastric administration of a marker solution (0.025 ml/g body weight) containing human serum albumin (50 mg/ml) and FITC-dextran 4000 (5.7 mg/ml). Four hours later the rats were deeply anesthetized by inhalation of isoflurane. Blood samples were taken by heart puncture into a syringe containing 1.5 mg EDTA and 20 KIU of a protease inhibitor (Trasylol, Bayer Health Care AG, Germany) and immediately ice chilled. Plasma was obtained after centrifugation (3000 × *g*, 15 min) and stored at − 80 °C until analysis. At autopsy thymus, spleen, pancreas, proximal and distal small intestine, and large intestine were dissected, weighted, and further processed for histochemistry and immunocytochemistry. In addition, fresh fecal samples were collected from the distal colon and stored at − 80 °C until analyzed for calprotectin.

The remaining three litters of totally 32 rats continued the experimental diets and were euthanized 4 weeks after start of experimentation. Food was removed 2–6 h before sacrifice and blood, organ, and faecal samplings were performed as described above. The numbers and gender distribution of rats in each group are listed in supplementary Table 6S.

### Tissue preparation

Tissue samples were fixed in 4% paraformaldehyde in 0.1 M phosphate buffer over night at 4 °C. The mid segment (≈2 cm long) of proximal and distal small and large intestine were opened along the mesenteric border, rinsed in saline and flattened on Whatman 3 mm chromatography paper before fixation. After fixation specimens were dehydrated in ethanol, cleared in xylene, orientated for longitudinal and cross sectioning (intestines) and embedded in paraffin. Sections (5 μm) were processed for hematoxylin–eosin (Htx-E) routine staining or immunocytochemistry.

### Immunocytochemistry

Sections were deparaffinized with xylene and rehydrated through graded series of ethanol followed by antigen retrieval by microwaving 2 × 8 min at 650 W in citrated acid buffer (0.01 M, pH 6). After cooling, slides were washed for 20 min in running tap water and 10 min in PBS with 0.25% Triton X-100 (PBS-T). Sections were incubated overnight in moist chamber at 4 °C with primary antibodies, rinsed in PBS-T buffer and incubated for 1 h with secondary antibodies. Details on antibodies used are given in Table 4. All antibodies were diluted in PBS-T containing 0.25% BSA.

**Table 4:** Details on primary and secondary antibodies used in immunocytochemistry.

Raised against	Code no	Host	Working dilution	Supplier
CD3	1127	Goat	1:1,000	Santa Cruz Biotechnology Inc., CA, USA
Proinsulin	9004	Guinea pig	1:2,000	Eurodiagnostica AB, Malmö, Sweden
Glucagon	7810	Rabbit	1:2,000	Eurodiagnostica AB, Malmö, Sweden
Glucagon-like peptide-1	A2	Rabbit	1:4,000	Kind gift from Prof R Ekman, Gothenburg, Sweden
Somatostatin	1758	Rabbit	1:1,600	Kind gift from Prof JJ Holst, Copenhagen, Denmark
Nkx6.1	NBP1-49,672	Rabbit	1:1,600	Novusbio, Abingdon, Oxford, UK
PDX-1/IPF1	AF2517	Goat	1:400	R&D Systems, Abingdgon, Oxford, UK
Guinea pig IgG	706 485 148	Donkey	1:1,500	Jackson ImmunoResearch, Europe Ltd, Sweden
Rabbit IgG	711 585 152	Donkey	1:1,500	Jackson ImmunoResearch, Europe Ltd, Sweden
Goat IgG	705 545 147	Donkey	1:1,200	Jackson ImmunoResearch, Europe Ltd, Sweden

To estimate the insulin:glucagon, insulin:GLP-1 and insulin:somatostatin cellular ratios in pancreatic islets sections were processed for double-immunolabelling using anti-insulin antibodies raised in guinea pig combined with antibodies raised in rabbit against glucagon, GLP-1 or somatostatin. Slides were then incubated with secondary antibodies raised in donkey against guinea pig IgG (Alexa Fluor488 conjugated), and rabbit IgG (Alexa Fluor594 conjugated). After washing sections were mounted in phosphate buffer:glycerol 1:1. Likewise anti-insulin antibodies raised in guinea pig and antibodies raised in rabbit against Nkx6.1 or in goat against PDX-1 were used in double-immunolabelling to study the cellular distributions of Nkx6.1 and PDX-1 in pancreatic islets.

To study numbers and topographic distribution of intestinal intraepithelial T cells and GLP-1 containing endocrine cells and their possible spatial relationship double-immunolabelling using the pan T-cells marker anti-CD3 antibodies raised in goat combined with antibodies raised in rabbit against GLP-1 followed by incubation with secondary antibodies raised in donkey against rabbit IgG (Alexa Fluor594 conjugated), and goat IgG (Alexa Fluor488 conjugated).

Antisera that had been inactivated by the addition of an excess amount of antigen (10–100 μg synthetic peptide/ml antiserum in working dilution) were used as controls. Further, the glucagon antiserum did not cross react with GLP-1 and the GLP-1 antiserum did not cross react with glucagon. Synthetic immunogens to the CD3, the Nkx6.1 and the PDX-1 antisera are not commercially available; therefore, omission of primary antibodies was used as control.

### Morphometric analysis

Htx-E and immunocytochemically stained sections from thymus, pancreas, proximal and distal small intestine and proximal large intestine were scanned on a digital scanner, Nanozoomer 2.0-HT, equipped with appropriate filters and NDP2-viewer software (Hamamatsu Photonics K.K., Japan; LRI Instrument AB, Lund Sweden).

In thymus and spleen, the numbers of apoptotic lymphocytes (identified as cells with pyknotic nuclei in Htx-E stained sections) were estimated per mm^2^ in thymic medulla and cortex and in spleen PALS. Ten randomly selected fields each 2500 μm^2^ were counted manually in each location in 4 rats from each dietary group.

In Htx-E stained sections from pancreas the total area of pancreatic tissue and the total area of pancreatic endocrine tissue (Langerhans islets) were determine. A minimum of 15 mm^2^ of total pancreatic area from at least two different depths was measured from each rat. Pancreatic islet size ranged from 90–101,000 μm^2^ and total islet area is expressed as percentage of total pancreatic area.

On sections from pancreas double immunolabelled for insulin and glucagon, GLP-1 or somatostatin 10 islets 3000–30,000 μm^2^ were selected from each rat and the numbers of insulin-, and glucagon-, GLP-1- or somatostatin- IR cells counted manually and the ratios of cells immunoreactive to insulin:glucagon, insulin:GLP-1 and insulin:somatostatin were determined.

In Htx-E stained sections from proximal and distal small intestine, and proximal large intestine measurements of layer thicknesses were performed on longitudinally and transversely cut intestinal whole wall sections. The mucosa, submucosa and muscularis propria, as well as whole wall thicknesses were defined using a binary cursor; mean values of 10 representative measurements were calculated from each rat.

On sections from distal small intestine and proximal large intestine double immunolabelled for CD3 and GLP-1 the numbers of CD3- and GLP-1-IR cells were estimated per mm epithelium in villus (small intestine), surface epithelium (large intestine) and crypts. The percentage of GLP-1-IR cells in contact, i.e. in close anatomic proximity, with CD3-IR cells were estimated by cell counting. Every 5th villi/surface epithelia and crypts were estimated, and 20 villi/surface epithelia and crypts were included per intestinal segment per rat.

### ELISA analysis

Insulin, C-peptide, leptin and IGF-1 levels were analyzed in blood plasma from rats after at least 2 h of fasting using commercially available ELISA kits according to the manufacturer’s instructions (insulin and C-peptide kits from Mercodia, Uppsala, Sweden and leptin and IGF-1 kits from R&D Systems, Minneapolis, MN, USA). All samples were run in duplicates and absorbance was measured at 450 nm in a FLOUstar Optima plate reader. Sample concentrations were estimated using a 7-point standard 4-parameter fit curve. The intra-coefficient of variation reached 5.2% for the insulin, 2.7% for the C-peptide, 2.8% for the leptin and 4.3% for the IGF-1 assays.

Faecal calprotectin was extracted with an extraction kit and measured with ELISA according to the manufacturer’s instructions (Immunodiagnostika, Gmbh, Eschelbronn, Germany).

### Statistical analysis

Normal distribution was estimated using D’Agostino-Persons test. Data where normal distribution was accepted is presented as mean ± SD, and differences between groups determined using one way ANOVA followed by Dunnett ´s multiple comparison test. Other was presented as median with interquartile range, and differences between groups was determined using Kruskal–Wallis followed by Dunn ´s multiple comparison test.

## Supplementary Information

Supplementary Information.

## Data Availability

All datasets generated and analysed are included in the published article or in its supplementary information files.
