# Supplementary material for: Structural and immunoendocrine remodeling in gut, pancreas and thymus in weaning rats fed powdered milk diets rich in Maillard reactants
Source: Sci Rep. 2022 Mar 8;12:4039. doi: 10.1038/s41598-022-08001-w (PMC8904556; doi:10.1038/s41598-022-08001-w)
Supplement: Supplementary file 1 — Supplementary Information. [file 41598_2022_8001_MOESM1_ESM.pdf]

Supplementary Tables

Structural and immunoendocrine remodeling in gut, pancreas and thymus in weaning rats fed powdered milk diets rich in Maillard reactants

J Dereke, E Ekblad, B Weström, C Erlanson-Albertsson, M Landin-Olsson, I Sjöholm and M Hillman

day	C	MM	p	HM	p
0	54.9±4.2	54.5±3.5	ns	54.5±4.2	ns
4	79.5±6.4	73.9±4.6	0.0201	73.1±5.8	0.0075
7	101.8±9.4	92.5±6.5	0.0053	90.8±7.3	0.0010
14	158.0±19.3	144.4±5.9	0.0410	139.9±10.8	0.0063
21	208.2±31.8	192.6±13.0	ns	187.3±18.6	ns
28	254.7±47.8	237.5±23.4	ns	221.9±33.7	ns

Supplementary Table 1S. Body weight (g) of rats weaned at 3 weeks of age (day 0) and fed control (C), medium Maillard (MM) or high Maillard (HM) containing diets for 0-28 days. Values are mean±SD, n = 14-15 (0-7 days), n = 10-11 (14-28 days). One-way ANOVA followed by Dunnett´s multiple comparison test. p= p-value as compared to C, ns= no significant differences compared to C.

	C	MM	HM	Group p-value
**0 - 7 days**	80.3 ± 6.7	86.2 ± 6.0	85.2 ± 8.0	0.467
**8 - 14 days**	122.8 ± 11.9	117.2 ± 19.8	112.6 ± 19.1	0.780
**15 - 21 days**	153.1 ± 10.3	132.0 ± 26.4	129.3 ± 17.8	0.325
**22 -28 days**	147.4 ± 13.0	134.1 ± 36.2	121.9 ± 25.8	0.542

Supplementary Table 2S. Feed intake (g) per week of rats fed control (C), medium Maillard (MM) or high Maillard (HM) containing diets for 4 weeks. Values are mean (of mean per cage) ± SD, n = 4 (0-7 days), n = 3 (8-28 days). Group p value determined by one-way ANOVA.

	C	MM	HM	Group p value
**1 week**				
Insulin (µg/L)	0.29 (0.24-0.39)	0.33 (0.24-0.43)	0.15 (0.15-0.18)	0.73
C-peptide (nmol/L)	0.45 (0.43-0.49)	0.41 (0.32-0.66)	0.34 (0.31-0.38)	0.14
Leptin (µg/L)	0.63 (0.46-0.79)	0.62 (0.51-0.80)	0.61 (0.51-0.79)	0.97
IGF-1 (µg/L)	434 (379-478)	297 (291-299)*	319 (307-322)	0.02
**4 weeks**				
Insulin (µg/L)	0.61 ± 0.23	0.56 ± 0.23	0.59 ± 0.23	0.93
C-peptide (nmol/L)	0.93 ± 0.25	0.82 ± 0.37	0.90 ± 0.33	0.71
Leptin (µg/L)	1.66 ± 0.68	1.61 ± 0.48	1.31 ± 0.74	0.47
IGF-1 (µg/L)	952 ± 78	887 ± 157	823 ± 118	0.10

SupplementaryTable 3S. Plasma levels of insulin, C-peptide, leptin and insulin-like growth factor-1 (IGF-1) in rats fed control (C), medium Maillard (MM) or high Maillard (HM) containing diets for 1 or 4 weeks. Values from rats fed 1 week are expressed as median (interquartile range), n=4, values from rats fed 4 weeks are expressed as mean ±SD or median (interquartile range), n = 10-11. P value determined by Kruskal-Wallis or one-way ANOVA. * p value= 0.02, compared to C, Kruskal-Wallis followed by Dunn´s multiple comparison test.

ID	Group	Insulin (µg/L)	C-peptid (nmol/L)	Leptin (µg/L)	IGF-1 (µg/L)	HSA (µg/L)	FD-4 (µg/L)	Calprotectin (µg/L)
1	C 4w	0.56	1.08	2.38	893			not detected
2	C 4w	0.99	1.07	0.82	907			not detected
3	C 4w	0.81	0.98	1.55	872			not detected
4	C 4w	0.72	0.85	1.39	916			0.11
5	MM 4w	0.17	0.45	1.57	720			
6	MM 4w	0.27	0.65	1.95	835			not detected
7	MM 4w	0.48	0.84	2.29	808			not detected
8	MM 4w	0.36	0.63	0.95	686			not detected
9	HM 4w	0.9	1.31	1.19	880			not detected
10	HM 4w	0.61	0.7	1.02	695			not detected
11	HM 4w	0.35	0.52	0.71	903			not detected
12	HM 4w	0.4	0.63	0.56	649			not detected
13	C 4w	0.18	0.65	1.42	1056			not detected
14	C 4w	0.32	0.5	1.16	914			not detected
15	MM 4w	0.71	0.98	1.74	1033			not detected
16	MM 4w	0.58	0.71	1.41	1163			0.48
17	MM 4w							
18	HM 4w	0.72	1.15	1.44	1021			not detected
19	HM 4w	0.41	0.76					0.07
20	HM 4w							
21	C 4w	0.73	1.38	3.18	890			not detected
22	C 4w	0.65	1.02	1.83	985			not detected
23	C 4w	0.52	0.78	1.22	1104			not detected
24	C 4w	0.59	1.05	1.69	987			not detected
25	MM 4w	1.3	1.44	2.24	995			not detected

26	MM 4w	1.14	1.45	1.24	940			not detected
27	MM 4w	0.34	0.59	1.13	804			not detected
28	MM 4w	0.21	0.45					0.1
29	HM 4w	0.8	1.2	2.19	804			not detected
30	HM 4w	0.76	1.25	2.6	834			not detected
31	HM 4w	0.2	0.4	0.74	796			not detected
32	HM 4w	0.71	1.07					0.04
33	C 1w	0.28	0.41	0.73	469	985.5	0.716	not detected
34	C 1w	0.19	0.46	0.53	399	157.3	0.168	0.11
35	C 1w	0.3	0.44	0.38	359	785.7	0.487	0.21
36	C 1w	0.47	0.51	0.85	486	59.2	0.502	0.33
37	MM 1w	0.33	0.3	0.64	300	35.5	0.54	0.05
38	MM 1w	0.52	0.85	0.6	285	38.6	0.525	0.32
39	MM 1w	0.16	0.47	0.95	296	52.8	0.493	0.04
40	MM 1w	0.32	0.34	0.41	297	678.1	0.492	0.23
41	HM 1w	0.15	0.35	0.88	324	4.91	0.553	not detected
42	HM 1w	0.15	0.32	0.53	318	124.9	0.648	not detected
43	HM 1w	0.15	0.29	0.48	320	159.2	0.476	not detected
44	HM 1w	0.21	0.4	0.69	295	4.91	0.72	not detected

SupplementaryTable 4S. Raw data of the individual rats (ID) after 1 or 4 weeks on control (C), medium Maillard (MM) or high Maillard (HM) diets from the ELISA analysis of insulin, C-peptide, leptin, insulin growth factor-1 (IGF-1), human serum albumin (HSA), FITC-dextran 4000 (FD-4) and calprotectin.

	C	MM	p-value	HM	p-value
HSA (µg/L)	471 (108-886)	46 (37-365)	ns	65 (5-142)	ns
FD-4 (ng/L)	495 (328-609)	509 (493-533)	ns	601 (515-684)	ns

Supplementary Table 5S. Plasma levels of the permeability markers human serum albumin (HSA) and FITC-dextran 4000 (FD-4) 4 hours after gavage in rats fed control (C), medium Maillard (MM) or high Maillard (HM) diets for 1 week. Values are median (interquartile range), n=4. Kruskal-Wallis followed by Dunn´s multiple comparison test. ns= no significant differences compared to C.

Table 6 supplement. Numbers and sex distribution of 1- and 4-week rats fed control (C), medium Maillard (MM) or high Maillard (HM) containing diets.

	C	MM	HM
**1-week**			
Males	4	3	3
Females	0	1	1
**4-weeks**			
Males	5	5	4
Females	5	6	7
